# The Baruch-Von Gyoery Discussion

**Published:** 1904-06

**Authors:** 


### THE BARUCH–VON GYOERY DISCUSSION.

SIMON BARUCH, of New York, and Dr. Von Gyoery, of Buda Pesth, are engaging in a discussion concerning the priority of Oliver Wendell Holmes over Semmelweis in the discovery of the infectious nature of puerperal sepsis. Baruch has shown, what was already well known, that to Holmes was due the honor of systematizing the knowledge of the causation of the condition, and using his literary skill in putting forth with great force the conception of its infectiousness and transmissibility. These results were published in 1843, at which time Semmelweis was a student of medicine. Great honor is due to Semmelweis for his practical, though for long unappreciated, work in the sanitation of child-bed, but the priority of the conception of the nature of that dreadful complication, puerperal fever, falls to Holmes.

This contention, though not so burdened with rival claimants, reminds one, from the date and the New England residence of one of the disputants, of the polemic waged over anesthesia. This was, for a long time, bitter and acrimonious in the extreme, and even now, still unsettled, spasmodically lights up. The New Englanders, with that tendency to magnify and glorify any product of their soil, were the principal champions of Morton's claims over Long, and those who were not for Morton swore allegiance to Wells, the Hartford dentist, whose laughing-gas was not all that made his contention a joke.

It is an easy matter for them (and for any one else) to see that Holmes' paper on puerperal fever was written and his views given in 1843 and that, therefore, he was the first in the field, and yet perception seems benumbed when it appears with equal simplicity and directness that Long used ether for surgical purposes in 1844, and Morton in 1846. Cold facts seem to chill the understanding.

